# 

**Published:** 1888-02

**Authors:** 


WHOLE NUMBER CCCXIX. | FEBRUARY, 1888. | NUMBER 7. VOLUME XXVII.

### THE BUFFALO Medical AND Surgical Journal

ESTABLISHED YEAR 1844.

LIBRARY INDEXED S.G.O.

**EDITORS:**

**THOS. LOTHROP, M. D.** **A. R. DAVIDSON, M. D.**

**ASSOCIATE EDITORS:**

**FLOYD S. CREGO, M. D.** **CARLTON C. FREDERICK, M. D.**
**FRED. R. CAMPBELL, M. D.** **FRANK H. POTTER, M. D.**

**EDWARD B. ANGELL, M. D., Rochester, N. Y.**

Entered at the Post-Office at Buffalo, N. Y., as Second-class Mail Matter.

